# Lactate levels during anesthesia in patients undergoing craniopharyngioma surgery

**DOI:** 10.3389/fsurg.2025.1541810

**Published:** 2025-06-10

**Authors:** Xiaoxu Shen, Xiaoyun Cao, Xuehua Che, Kaiyu Wang, Nidan Qiao

**Affiliations:** ^1^ Department of Nursing, Shanghai Medical School, Huashan Hospital, Fudan University, Shanghai, China; ^2^ Department of Neurosurgery, Shanghai Medical School, Huashan Hospital, Fudan University, Shanghai, China; ^3^ National Center for Neurological Disorders, Shanghai, China; ^4^ Shanghai Clinical Medical Center of Neurosurgery, Shanghai, China; ^5^ Neurosurgical Institute of Fudan University, Shanghai, China; ^6^ Shanghai Key Laboratory of Medical Brain Function and Restoration and Neural Regeneration, Fudan University, Shanghai, China; ^7^ Department of Anesthesia, Shanghai Medical School, Huashan Hospital, Fudan University, Shanghai, China

**Keywords:** electrolyte disturbance, neurosurgery, anesthesia, craniopharyngioma, lacate levels

## Abstract

**Purpose:**

To assess the incidence of hyperlactatemia and lactic acidosis in patients undergoing craniopharyngioma surgery and to investigate their association with surgical outcomes.

**Method:**

We analyzed clinical data from all patients who underwent craniopharyngioma surgery between 2019 and 2023 at a tertiary care center. Arterial blood gas analyses were performed prior to anesthesia and at one-hour intervals during surgery. Patients were classified into three groups: the Lactic Acidosis group (LA), Hyperlactatemia group (HL), and Normal Control group (NC). The primary outcome was the occurrence of postoperative severe hypernatremia (serum sodium levels exceeding 155 mmol/L).

**Results:**

We enrolled 261 patients with a mean age of 41.7 years. During anesthesia, mean lactate levels increased from 1.4 [1.0–1.9] mmol/L at initiation to 4.6 [1.8–7.0] mmol/L after 8 h. Among the cohort, 44 patients (16.9%) were classified in the HL group and 31 patients (11.9%) in the LA group. Anesthesia duration was the sole factor significantly associated with increased lactate levels in both univariate and multivariable analyses (OR 1.50 [95% CI: 1.31–1.80], *p* < 0.001). The elevated lactate level was independently associated with hypernatremia, even after adjusting for potential confounders, with an odds ratio of 2.12 (95% CI: 1.04–4.24, *p* = 0.038). No significant differences were observed among the three groups regarding total hospital stay, ICU stay, or incidence of severe complications.

**Conclusion:**

Lactate levels increased during anesthesia in patients undergoing craniopharyngioma surgery and were associated with postoperative hypernatremia. However, with appropriate management, lactic acidosis was not significantly linked to adverse postoperative outcomes.

## Introduction

Craniopharyngioma is an intracranial neoplasm that arises from remnants of the craniopharyngeal duct, typically located in the hypothalamic-pituitary region. Due to its proximity to critical structures such as the pituitary gland, pituitary stalk, and optic chiasm, its management poses significant challenges. Neurosurgery, either via transcranial or transsphenoidal approaches, remains the primary treatment modality (1). However, surgical intervention is associated with a considerable risk of major adverse events (2, 3), with an overall mortality rate ranging from 2% to 3% (4). One of the most frequent postoperative complications is diabetes insipidus (DI) (5), which can result in hypoperfusion and hypernatremia (6, 7).

Diabetes insipidus is characterized by excessive thirst and dilute polyuria, primarily caused by defects in arginine vasopressin synthesis, secretion, or renal response. Current classifications delineate four subtypes based on etiology: central DI, nephrogenic DI, primary polydipsia and gestational DI.

Mechanical manipulation of the pituitary stalk during surgery can trigger central diabetes insipidus immediately, even under anesthesia. If fluid replacement during surgery is insufficient, hypoperfusion can occur, potentially leading to elevated lactic acid levels. These elevated levels may persist post-anesthesia and continue for several days after surgery. However, the relationship between hyperlactatemia and outcomes following craniopharyngioma surgery remains controversial. Normal blood lactate levels are typically defined as concentrations below 2.0 mmol/L, but no universally accepted threshold for hyperlactatemia exists, with values above 3 mmol/L, 4 mmol/L, or even 10 mmol/L being variably classified (8, 9). Severe hyperlactatemia can progress to lactic acidosis, a condition linked to poor tissue perfusion and multiorgan dysfunction. In hospital settings, lactic acidosis is a recognized prognostic marker for mortality, particularly in patients with severe sepsis and septic shock (10, 11).

This study aimed to determine the incidence of hyperlactatemia and lactic acidosis in patients with craniopharyngioma undergoing surgery. Additionally, it sought to explore the association between the occurrence of hyperlactatemia, lactic acidosis, and surgical outcomes. The findings of this study could provide valuable insights into improving perioperative management and optimizing outcomes for patients with craniopharyngioma.

## Materials & methods

### Study design and participants

This retrospective study analyzed clinical data from all patients who underwent craniopharyngioma surgery between 2019 and 2023 at a tertiary care center. Ethical approval was granted by the local Institutional Review Board (IRB), and the study adhered to the principles outlined in the 1975 Helsinki Declaration. Written informed consent was obtained from all patients upon data collection. Inclusion criteria were patients with craniopharyngioma treated surgically. Exclusion criteria included: (1) administration of ${\mathrm{NaHCO}}_{3}^{-}$, (2) emergency surgeries, (3) chronic renal insufficiency requiring dialysis, and (4) preoperative acute or chronic liver failure.

All patients underwent a standardized endocrine assessment prior to surgery at our center. Central adrenal insufficiency was defined by a morning cortisol level of less than 3 mg/dl, while levels above 15 mg/dl were considered normal. For patients with morning cortisol levels between 3 and 15 mg/dl, further testing was performed using either an adrenocorticotropic hormone (ACTH) stimulation test or an insulin tolerance test. A peak cortisol value below 18 mg/dl was indicative of central adrenal insufficiency. Central hypothyroidism was diagnosed based on low serum free thyroxine levels and insufficient thyroid hormone levels. To diagnose central diabetes insipidus, clinical presentation, urine specific gravity, urine and serum osmolality, serum sodium levels, and the need for desmopressin treatment were comprehensively evaluated.

For patients undergoing transnasal resection of craniopharyngiomas, anesthesiologists are not required to maintain relatively low intracranial pressure, as cerebrospinal fluid (CSF) is released during the transnasal resection of the tuberculum dura. Therefore, the use of osmotically active drugs is unnecessary and was not employed in these cases. All patients were managed using an inhalation anesthesia technique.

### Data collection and definitions

The following data were collected: baseline patient characteristics prior to surgery, intraoperative findings, postoperative blood test results, and complications. Arterial blood gas analyses were conducted before anesthesia and at one-hour intervals during anesthesia using an automated analyzer (ABL90, Radiometer Medical Aps, Bronshoj, Denmark).

Patients were categorized into three groups based on their highest recorded blood lactate levels and lowest pH measurements:

• **Lactic Acidosis Group (LA)**: blood lactate levels ≥ 5 mmol/L with pH < 7.30.
• **Hyperlactatemia Group (HL)**: blood lactate levels ≥ 5 mmol/L with pH ≥ 7.30 and <7.50.
• **Normal Control Group (NC)**: blood lactate levels < 5 mmol/L with pH ≥ 7.30 and <7.50.

The postoperative steroid regimen was standardized across the cohort, consisting of intravenous methylprednisolone followed by oral cortisone. Serum electrolytes were monitored every morning after surgery, and for patients with severe hypernatremia, additional measurements were taken in the afternoon when necessary. Hypernatremia was managed based on the serum sodium levels, using saline, hypotonic saline, or oral water as appropriate.

### Outcomes

For the first study aim, the primary outcome was postoperative severe hypernatremia, defined as a serum sodium level exceeding 155 mmol/L. The duration of consecutive days with hypernatremia was documented for each patient. Multivariable logistic regression was employed to identify potential risk factors for severe hypernatremia. The predictors assessed included patient demographics (age, sex), presenting symptoms (headache, visual disturbances, polyuria, weakness), tumor characteristics (diameter, type: intrasellar, suprasellar, or third ventricular), calcification status, and surgical findings (approach, stalk preservation, and extent of resection). Additionally, pre anesthesia blood gas variables such as PH, hemoglobin, hematocrit, glucose, potassium, sodium, HCO3−, anion gap and base excess were included in the analysis. To minimize bias during data entry, investigators were blinded to both the predictors and outcomes using natural language processing and automatic data extraction techniques.

Other secondary outcomes included the number of consecutive days with hypernatremia, highest postoperative urine volume, and lowest postoperative serum sodium levels. Additionally, we compared severe complications, postoperative ICU stay duration, and overall hospital length of stay among the three groups. Severe complications were defined as intracranial hemorrhage, cerebral edema, ischemic stroke, hypothalamic dysfunction, or any complication leading to death or coma (i.e., a Glasgow Coma Score < 13). These outcomes were assessed to provide a comprehensive evaluation of postoperative recovery and morbidity.

### Statistical analysis

The statistical analysis was performed using R software version 3.4.2. Continuous variables were expressed as means with standard deviations or medians with interquartile ranges. Continuous variables between two groups were analyzed using *t*-test or Mann–Whitney test, and comparisons between groups were made using ANOVA with a *post-hoc* test. Categorical variables were presented as frequencies (*n*, %) and analyzed using the chi-square test. Multivariable logistic regression analysis was conducted to identify factors associated with elevated lactate levels.

## Results

### Clinical characteristics of the patients

The study enrolled 261 patients with a mean age of 41.7 years, of whom 54% were male. Recurrent tumors were present in 20.7% of the patients. The majority of the patients were treated using a transsphenoidal approach (see Table 1). Total tumor resection was achieved in 68.2% of the cases, while only 34.1% of the patients underwent pituitary stalk preservation.

**Table 1:** Characteristics of the cohort.

Characteristics	All	Normal control	Hyperlactatemia	Lactic acidosis	*p*
	(*N* = 261)	(NC, *N* = 186)	(HL, *N* = 44)	(LA, *N* = 31)	
Age (years old)	41.7 (17.6)	40.4 (17.9)	47.0 (17.2)	42.3 (16.6)	0.083
Sex (male)	141 (54.0%)	92 (49.5%)	28 (63.6%)	21 (67.7%)	0.063
BMI	23.9 (3.8)	23.7 (3.9)	24.5 (3.8)	24.2 (3.9)	0.484
Surgical history	54 (20.7%)	36 (19.4%)	12 (27.3%)	6 (19.4%)	0.497
Symptoms					
Headache	66 (25.3%)	49 (26.3%)	7 (15.9%)	10 (32.3%)	0.228
Visual disturbance	128 (49.0%)	87 (46.8%)	24 (54.5%)	17 (54.8%)	0.514
Polyuria and hypopituitary	68 (26.1%)	50 (26.9%)	9 (20.5%)	9 (29.0%)	0.630
Radiological characteristics					
Calcification	148 (59.1%)	113 (60.8%)	24 (54.5%)	11 (35.5%)	0.030
Cystic formation	207 (81.8%)	148 (79.6%)	37 (84.1%)	22 (71.0%)	0.380
3rd Ventricular type	32 (10.6%)	20 (10.8%)	6 (13.6%)	6 (19.4%)	0.383
Surgery					
Transsphenoidal approach	250 (95.8%)	178 (95.7%)	42 (95.5%)	30 (96.8%)	0.956
Total resection	178 (68.2%)	133 (71.5%)	29 (65.9%)	16 (51.6%)	0.083
Stalk preserved	89 (34.1%)	68 (36.6%)	9 (20.5%)	12 (38.7%)	0.109
Anesthesia duration (hours)	5.5 (2.1)	5.1 (1.9)	6.7 (2.1)	6.7 (1.8)	<0.001
Blood gas before anesthesia					
pH	7.4 (0.0)	7.4 (0.0)	7.4 (0.0)	7.4 (0.0)	0.088
Hemoglobin (g/dl)	12.8 (1.8)	12.7 (1.8)	12.9 (1.4)	13.1 (2.2)	0.527
Hematocrit (%)	39.3 (5.6)	39.0 (5.6)	39.6 (4.3)	40.1 (6.7)	0.538
Glucose (mmol/L)	5.4 (0.8)	5.4 (0.8)	5.7 (1.0)	5.3 (0.8)	0.055
K+ (mmol/L)	3.5 (0.3)	3.5 (0.3)	3.6 (0.3)	3.6 (0.2)	0.176
Na+ (mmol/L)	143.0 (4.1)	143.0 (3.8)	143.1 (5.1)	143.2 (4.2)	0.950
${\mathrm{HCO}}_{3}^{-}$ (mmol/L)	25.3 (2.3)	25.4 (2.2)	24.9 (2.5)	25.6 (2.3)	0.368
Anion Gap (mmol/L)	11.9 (2.0)	11.8 (1.8)	12.2 (2.0)	12.1 (2.9)	0.392
Base excess (mmol/L)	0.9 (2.4)	1.0 (2.3)	0.4 (2.7)	1.0 (2.6)	0.300

Continuous variables were analyzed using ANOVA, categorical variables were analyzed using the chi-square test.

### Lactate levels during anesthesia

**Figure 1:**
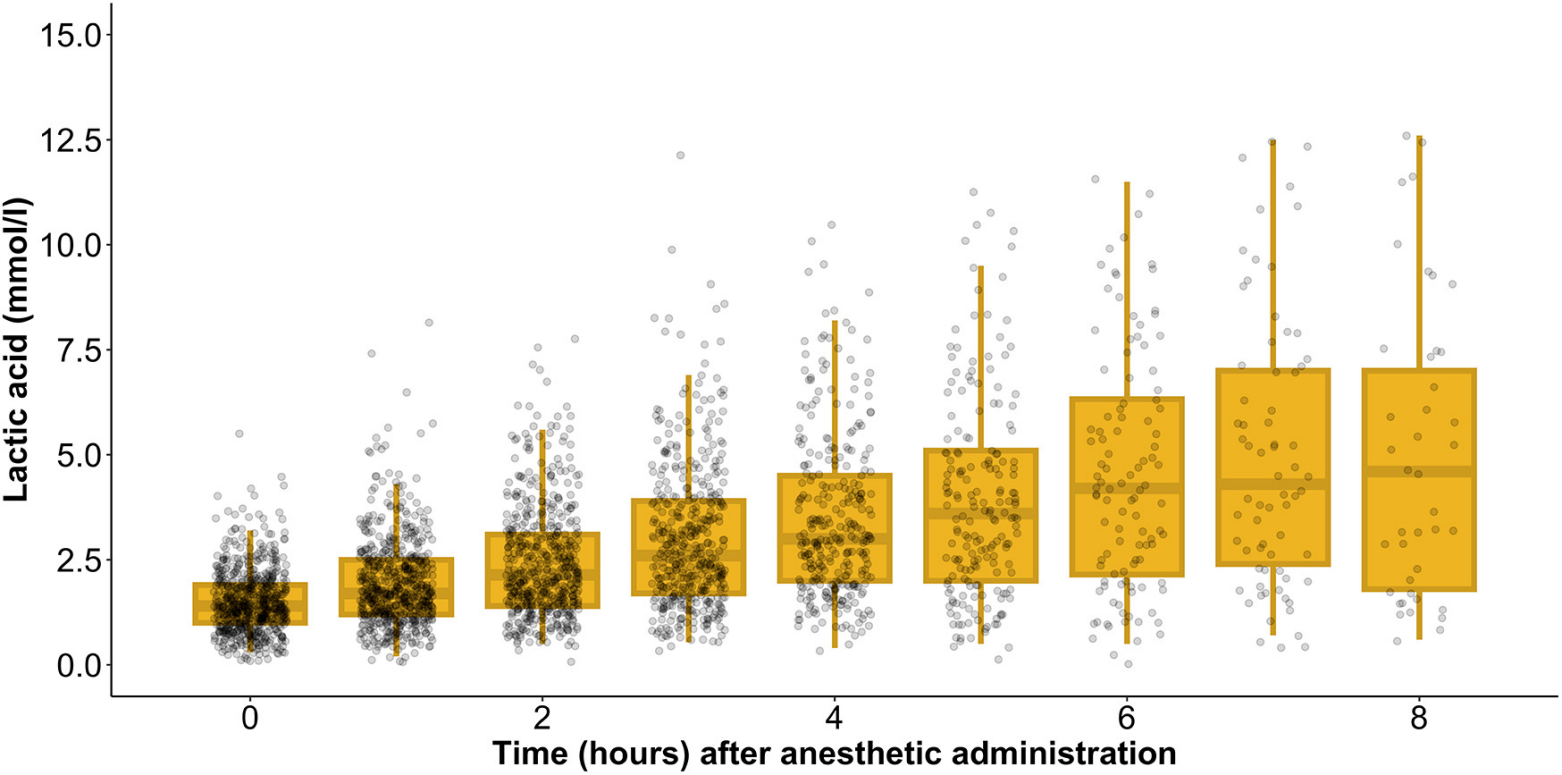
Gradual change of mean lactate level during anesthesia.

**Figure 2:**
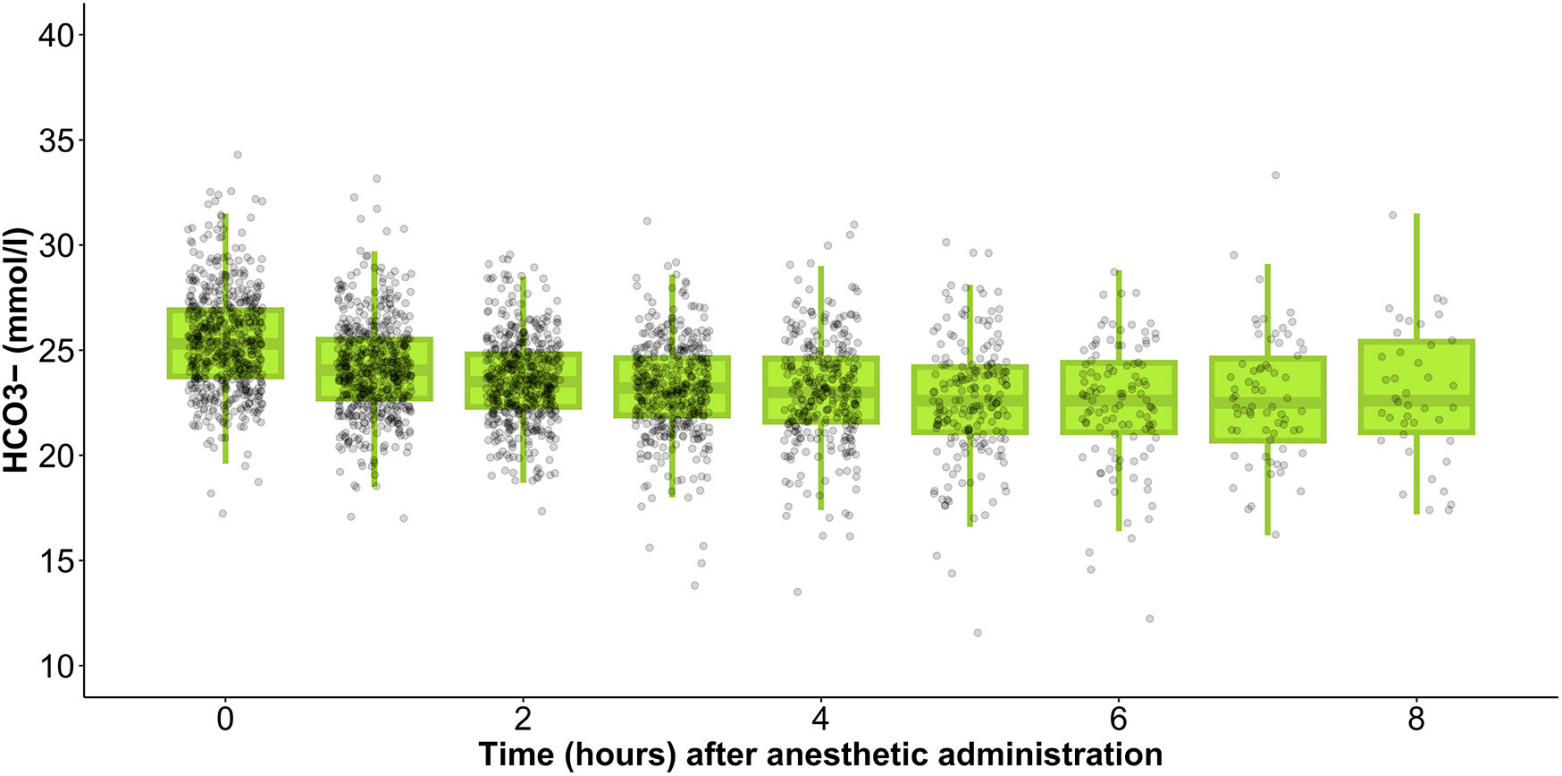
Gradual change of mean HCO3− level during anesthesia.

**Figure 3:**
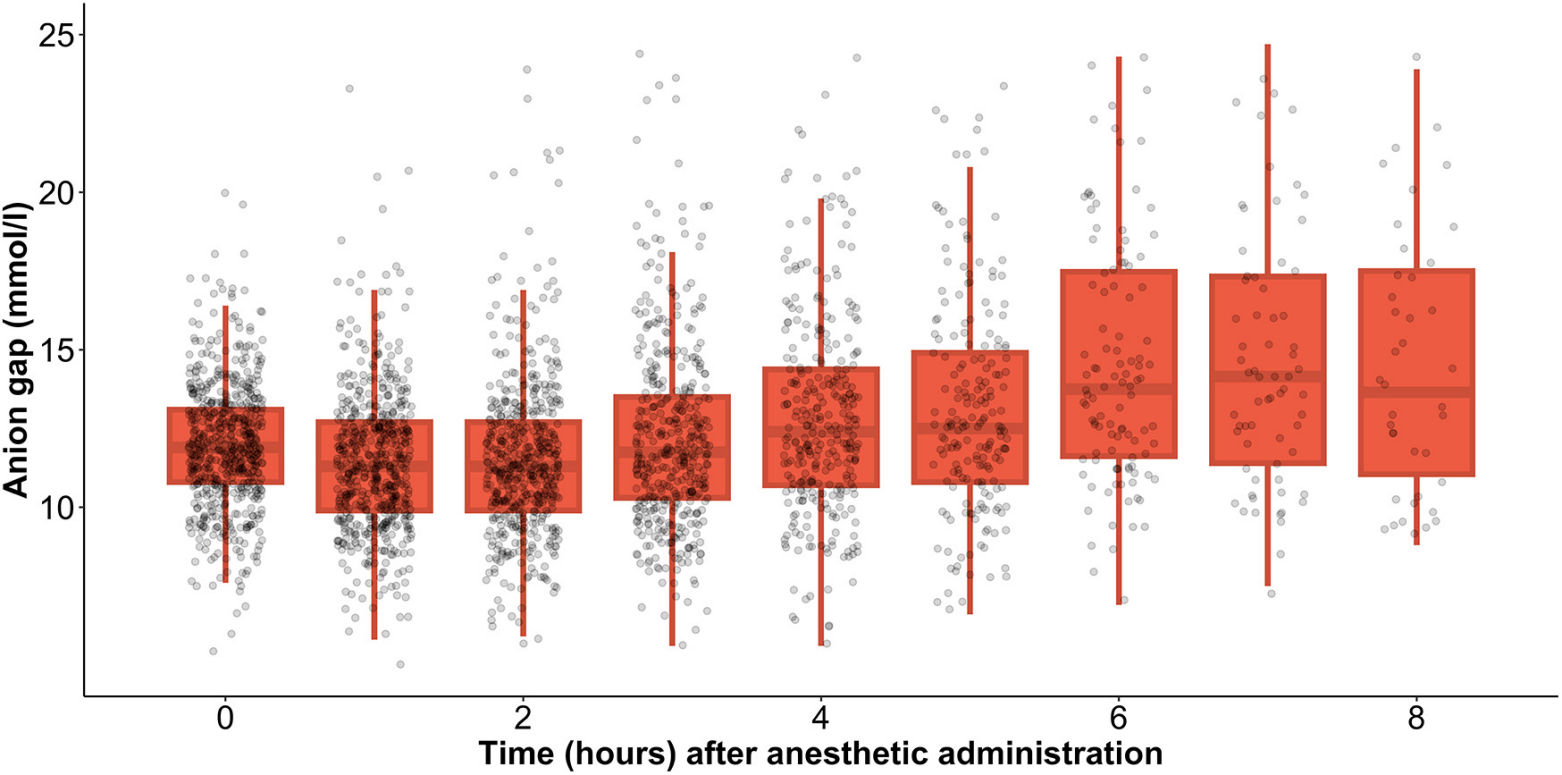
Gradual change of mean anion gap during anesthesia.

During anesthesia, the mean lactate level showed a gradual increase, starting from 1.4 [1.0–1.9] mmol/L at the initiation of anesthesia and rising to 4.6 [1.8–7.0] mmol/L after 8 h (Figure 1). Simultaneously, HCO3− levels decreased from 25.3 [23.8–26.9] mmol/L at the onset of anesthesia to 22.6 [21.1–25.4] mmol/L by the 8 h mark (Figure 2). In parallel, the anion gap increased from 12.0 [10.7–13.1] to 13.8 [11.2–17.5] during the same timeframe (Figure 3). Spearman’s correlation analysis revealed strong correlations among the three parameters, with all pairwise comparisons showing *p* < 0.001. These changes reflect metabolic alterations occurring during prolonged anesthesia.

However, the number of observations varied across time points, as some patients had shorter surgeries and, consequently, shorter anesthetic durations. To account for this, we analyzed individual time-dependent changes in each patient and found that 94.6% exhibited an increasing lactate trend. Additionally, 40.6% had a lactate increase exceeding 2 mmol/L, while 10.3% experienced an increase greater than 5 mmol/L.

### Factor associated with increased level of lactate

Within the cohort, 186 patients (71.2%) maintained lactic acid levels below 5 mmol/L during anesthesia and were classified in the normal control group. Meanwhile, 44 patients (16.9%) exhibited lactic acid levels above 5 mmol/L but preserved a normal pH, placing them in the hyperlactatemia group. The remaining 31 patients (11.9%) had lactic acid levels exceeding 5 mmol/L accompanied by a decreased pH level, classifying them in the lactic acidosis group.

In the univariate analysis, baseline pH, HCO3−, and lactate levels were similar across all three groups, with no statistically significant differences (all *p* > 0.05). Anesthesia duration emerged as the only factor associated with increased lactate levels in the univariate analysis (Table 1). In the subsequent multivariable analysis, anesthesia duration remained a significant predictor of elevated lactate levels. Specifically, the odds of developing hyperlactatemia increased by 1.3 times for every additional hour of anesthesia, while the odds of developing lactic acidosis increased by 1.2 times per additional hour of anesthesia (Table 2). These findings underscore the critical role of anesthesia duration in influencing lactate dynamics during surgery.

**Table 2:** Multivariable analysis identifying risk factors associated with HL and LA.

	Odds ratio	*p*
HL+LA vs. NL		
Anesthesia duration	1.50 [1.31–1.80]	<0.001
LA vs. HL+NL		
Anesthesia duration	1.33 [1.09–1.62]	0.005

### Outcomes

In patients who developed hyperlactatemia and lactic acidosis during surgery, normalization of lactic acid and pH levels occurred within one to two days postoperatively. This rapid correction was achieved through prompt and adequate volume replenishment.

When comparing the primary outcomes among the three groups (Table 3), the proportion of patients who developed hypernatremia with Na+ more than 155 mmol/L did not reach statistical significance, although the *p*-value was 0.055. Notably, a slightly higher number of patients in the LA group developed hypernatremia compared to the normal NC group. In terms of secondary outcomes, the peak serum sodium levels were comparable across all three groups, as was the number of consecutive days with hypernatremia. However, the highest urine volume was significantly elevated in both the HL and LA groups compared to the NC group. There were no significant differences between the groups in total hospital stay, ICU stay, or the incidence of severe complications.

**Table 3:** Outcomes in the three groups.

Outcomes	Normal control	Hyperlactatemia	Lactic acidosis	*p*		
	(NC, *N* = 186)	(HL, *N* = 44)	(LA, *N* = 31)	Three groups comparison	HL vs. HC	LA vs. NC
Primary outcome						
Hypernatremia with Na+ >155 mmol/L	45 (24.2%)	16 (36.4%)	13 (41.9%)	0.055	0.146	0.065
Other outcomes						
Highest sodium level (mmol/L)	150.8 (6.7)	151.2 (6.8)	152.7 (5.7)	0.250	0.799	0.193
Lowest sodium level (mmol/L)	134.2 (6.2)	133.6 (4.9)	133.6 (5.9)	0.744	0.774	0.831
More than 5 continuous days with hypernatremia	11 (5.9%)	3 (6.8%)	4 (12.9%)	0.364	1.000	0.299
Highest urine volume (ml)	5,414 (1,279)	5,699 (1,357)	6,167 (1,418)	0.010	0.347	0.007
Post-operative stays (Days)	13.0 (9.9)	13.8 (8.9)	13.3 (7.7)	0.876	0.848	0.980
ICU stays (Days)	1.2 (0.7)	1.6 (2.3)	1.3 (0.7)	0.114	0.076	0.997
Severe complications	7 (3.8%)	2 (4.5%)	1 (3.2%)	0.954	1.000	1.000

Continuous variables were analyzed using ANOVA, categorical variables were analyzed using the chi-square test.

When the HL and LA groups were combined (Table 4), the proportion of patients who developed hypernatremia with Na+ more than 155 mmol/L reached statistical significance, with a *p*-value of 0.028. Specifically, 38.7% of patients in the HL and LA group experienced hypernatremia, compared to 24.2% in the normal control NC group. Other outcomes remained similar to the results observed in the three-group comparison. In the multivariable analysis, elevated lactate levels remained independently associated with the occurrence of hypernatremia, even after adjusting for potential confounders, with an odds ratio of 2.12 (95% CI: 1.04–4.24, *p* = 0.038).

**Table 4:** Outcomes in the two groups comparison.

Outcomes	Normal control	Hyperlactatemia + Lactic acidosis	*p*
	(*N* = 186)	(*N* = 44)	
Primary outcome			
Hypernatremia with Na+ >155 mmol/L	45 (24.2%)	29 (38.7%)	0.028
Secondary outcomes			
Highest sodium level (mmol/L)	150.8 (6.7)	151.83 (6.4)	0.167
Lowest sodium level (mmol/L)	134.2 (6.2)	133.57 (5.3)	0.441
More than 5 continuous days with hypernatremia	11 (5.9%)	7 (9.3%)	0.474
Highest urine volume (ml)	5,414 (1,279)	5,893 (1,392)	0.008
Post-operative stays (Days)	13.0 (9.9)	13.6 (8.4)	0.639
ICU stays (Days)	1.2 (0.7)	1.5 (1.9)	0.127
Severe complications	7 (3.8%)	3 (4.0%)	1.000

Continuous variables were analyzed using *t*-test or Mann–Whitney test, categorical variables were analyzed using the chi-square test.

## Discussion

We demonstrated the time-dependent changes in lactate acid levels, HCO3− and anion gap during anesthesia and after surgery in patients with craniopharyngioma. Lactate acid levels were found to be associated with the duration of anesthesia. Elevated lactate levels could be used as predictors for hypernatremia after surgery. On the contrary, although lactic acidosis occurred in approximately 10% of the patients, it was not linked to poor postoperative outcomes. These findings suggest that proper management is crucial in these patients to mitigate any potential adverse effects.

Lactate is traditionally viewed as a marker of tissue hypoxia and poor perfusion, and elevated levels are frequently linked to adverse outcomes in critically ill patients, particularly those with conditions such as sepsis or septic shock (12, 13). In the context of craniopharyngioma surgery, hyperlactatemia may reflect transient metabolic changes induced by anesthesia, surgical manipulation, and fluid management. It is well-established that mechanical disruption of the pituitary stalk or hypothalamic structures during surgery can lead to endocrine dysfunctions, including diabetes insipidus, which may, in turn, influence fluid balance and electrolyte abnormalities. Our study confirms that hyperlactatemia during surgery is time-dependent and is more likely to occur in patients with longer anesthesia durations. This finding emphasizes the need for careful monitoring of lactate levels throughout the perioperative period, particularly for patients with prolonged surgeries.

Interestingly, we found that lactic acidosis, although a recognized indicator of poor tissue perfusion, did not correlate with worse outcomes such as mortality, neurological deficits, or prolonged ICU stays in our cohort. This finding challenges the conventional view that lactic acidosis is an unequivocal sign of poor prognosis. For instance, Naik et al. reported an incidence of hyperlactatemia (blood lactate levels ≥ 4 mmol/L) in 42.7% of cardiovascular surgery patients, with these patients exhibiting higher rates of postoperative atrial fibrillation and longer ICU stays (14). Similarly, Renew et al. observed that severe hyperlactatemia (blood lactate levels > 10 mmol/L) was associated with increased mortality (15).

One potential explanation for this discrepancy could be the transient nature of the metabolic disturbance in craniopharyngioma surgery. Unlike in patients underwent cardiovascular surgery or with sepsis, where lactic acidosis reflects ongoing organ dysfunction, in the perioperative craniopharyngioma population, lactic acidosis may be more related to temporary alterations in circulatory and fluid balance that resolve with proper postoperative care.

Another important finding of our study was the association between urine volume and lactic acidosis post-surgery. Lactic acidosis may potentially exacerbate renal function disturbances (16) or contribute to electrolyte imbalances, further complicating the clinical management of patients postoperatively. While hypernatremia and diabetes insipidus are common complications after craniopharyngioma surgery, the relationship between fluid output, lactate levels, and renal function remains complex. This underscores the importance of a comprehensive and multidisciplinary approach to patient management that addresses both the metabolic and endocrine aspects of recovery. Patients who experience prolonged hypernatremia or require additional interventions, such as desmopressin administration, may benefit from closer monitoring of lactate levels as part of their routine postoperative care.

Altogether, these results suggest that, with adequate perioperative management—particularly fluid resuscitation, monitoring of electrolytes, and addressing hormonal imbalances—patients can recover without significant long-term detriment. This is particularly relevant for craniopharyngioma surgery, where the surgical approach and postoperative management play pivotal roles in determining recovery. The transient nature of metabolic disturbances like lactic acidosis in this context may not warrant aggressive intervention but rather close observation and supportive care.

This study has several limitations. First, being a retrospective analysis, it is inherently subject to biases associated with the available data, and the sample size was relatively small. The limited sample size likely contributed to the wide 95% confidence intervals observed in the regression analysis, which may have affected the precision of the associations between the predictors and outcomes. The study was conducted at a single center, which may limit the generalizability of the findings to other institutions with different patient populations, surgical practices, or management protocols.

Second, we did not collect statistics comparing goal-directed fluid therapy to a liberal fluid management strategy during anesthesia, as the choice of approach was at the discretion of the anesthesiologists. all patients were managed using an inhalation anesthesia technique. All patients were managed using an inhalation anesthesia technique. The use of an intravenous technique with propofol could mitigate lactate production compared to an inhalational technique and highlighted the need for further research on these topics (8, 17).

Third, Spiegelberg’s study (9) showed that lactate clearance within 12 h is a good predictor for the outcome. In their study, lactate clearance was calculated by comparing blood lactate levels 12 h after the first measurement exceeding 10 mmol/L. However, in our cohort, only a few patients had lactate levels above this threshold, making lactate clearance calculations insufficient. Moreover, the study did not comprehensively examine fluid management protocols or their potential influence on lactate dynamics during and after surgery. Given the significant role that fluid management may play in lactate metabolism, a more detailed exploration of this factor could provide valuable insights in future studies.

Finally, the focus of the study was primarily on immediate postoperative outcomes, such as hyperlactatemia, ICU stay, and hospital length of stay. However, the long-term effects of elevated lactate levels on functional recovery, quality of life, and neurological outcomes following surgery were not addressed, and further investigation is warranted.

In conclusion, lactate levels increased during anesthesia in patients undergoing craniopharyngioma surgery and were associated with postoperative hypernatremia. However, with appropriate management, lactic acidosis was not significantly linked to adverse postoperative outcomes.

## Data Availability

The raw data supporting the conclusions of this article will be made available by the authors, without undue reservation.
